# Unilateral aplasia of both cruciate ligaments

**DOI:** 10.1186/1749-799X-5-11

**Published:** 2010-02-25

**Authors:** Maurice Balke, Jonas Mueller-Huebenthal, Sven Shafizadeh, Dennis Liem, Juergen Hoeher

**Affiliations:** 1 Department of Trauma and Orthopedic Surgery, University of Witten-Herdecke, Cologne Merheim Medical Center, Ostmerheimerstrasse 200, 51109 Cologne, Germany; 2 Department of Radiology, Praxis im KoelnTriangle, Ottoplatz 1, 50679 Cologne, Germany; 3 Department of Orthopedic Surgery, University Hospital Muenster, Albert-Schweitzer-Str. 33, 48149 Muenster, Germany; 4 Division of Sports Medicine, Trauma Department, Hospital Cologne Merheim, University of Witten-Herdecke, Ostmerheimerstrasse 200, 51109 Cologne, Germany

## Abstract

Aplasia of both cruciate ligaments is a rare congenital disorder. A 28-year-old male presented with pain and the feeling of instability of his right knee after trauma. The provided MRI and previous arthroscopy reports did not indicate any abnormalities except cruciate ligament tears. He was referred to us for reconstruction of both cruciate ligaments. The patient again underwent arthroscopy which revealed a hypoplasia of the medial trochlea and an extremely narrow intercondylar notch. The tibia revealed a missing anterior cruciate ligament (ACL) footprint and a single bump with a complete coverage with articular cartilage. There was no room for an ACL graft. A posterior cruciate ligament could not be identified. The procedure was ended since a ligament reconstruction did not appear reasonable. A significant notch plasty if not a partial resection of the condyles would have been necessary to implant a ligament graft. It is most likely that this would not lead to good knee stability. If the surgeon would have retrieved the contralateral hamstrings at the beginning of the planned ligament reconstruction a significant damage would have occurred to the patient. Even in seemingly clear diagnostic findings the arthroscopic surgeon should take this rare abdnormality into consideration and be familiar with the respective radiological findings. We refer the abnormal finding of only one tibial spine to as the "dromedar-sign" as opposed to the two (medial and a lateral) tibial spines in a normal knee. This may be used as a hint for aplasia of the cruciate ligaments.

## Background

Aplasia of the cruciate ligaments is a very rare congenital pathology which was first described in 1956 by Giorgi as part of a radiographic study [1]. It is typically associated with other congenital musculoskeletal disorders such as absent radius syndrome [2], congenital meniscal malformations [3-5] and most commonly with longitudinal deficiencies of the lower limbs (e.g. congenital short femur, and aplasia of the fibula or patella) [6-10]. Malformations of the cruciate ligaments can either affect the anterior cruciate ligament (ACL) only or both cruciate ligaments [11-15]. The deficiency can occur unilaterally [4,5,7,9,16-20] or affect both knee joints [6,13,14,17]. We report on a patient with unilateral aplasia of both cruciate ligaments and point out the diagnostic pitfalls that possibly lead to therapeutic mistakes.

## Case presentation

**Figure 1:**
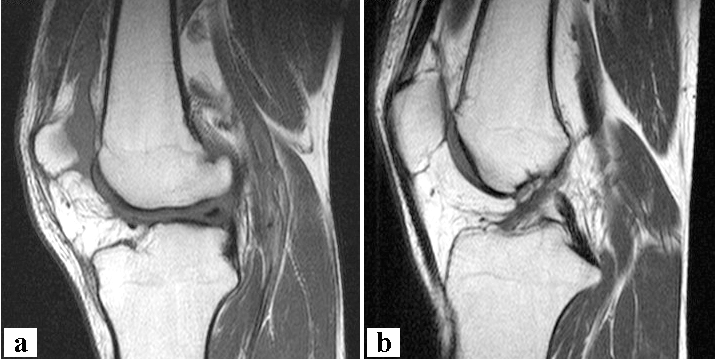
**Magnetic resonance imaging**. Sagittal T1 TSE sequences of the affected (**a**) and the contralateral (**b**) knee. Note the lack of both cruciate ligaments and the abnormal tibial eminence.

A 28-year-old white male presented in our office with knee problems after he hit a gym wall with his right knee during sports. His major complaint was anterior knee pain and the feeling of instability. He already underwent MRI (Figure 1) examination with the following report: "Complete, chronic tear of the anterior and posterior cruciate ligaments and chondropathia of the medial femoral condyle." No further abnormal findings were documented. He further was treated by diagnostic arthroscopy elsewhere with the following report: "Normal findings of the medial and lateral menisci, narrow notch with lack of an anterior cruciate ligament (ACL), insufficiency of the posterior cruciate ligament (PCL), chondropathia of the medial femoral condyle." Again no other abnormal findings were documented. The patient was now referred to our clinic for combined reconstruction of the anterior and posterior cruciate ligaments.

On clinical exam he had a free range of motion, no swelling and a slight valgus alignment. He had a positive posterior sag at 90° of flexion and a reduced medial step off when compared to the other side. His Lachman test was severely abnormal without a firm endpoint, pivot shift was slightly positive. His total anteroposterior laxity when measured with the Rolimeter (Aircast, Don Joy, Inc) was 6 mm and 22 mm with a resulting side difference of 16 mm. His collateral ligaments were stable. His further history revealed a status post medial growth plate closure at the medial femoral condyle at the age of 12 for a significant leg length discrepancy and a syndactylia of the second and third toe of his right foot.

Due to the clinical, surgical and MRI findings the patient was scheduled to undergo ACL and PCL reconstruction.

**Figure 2:**
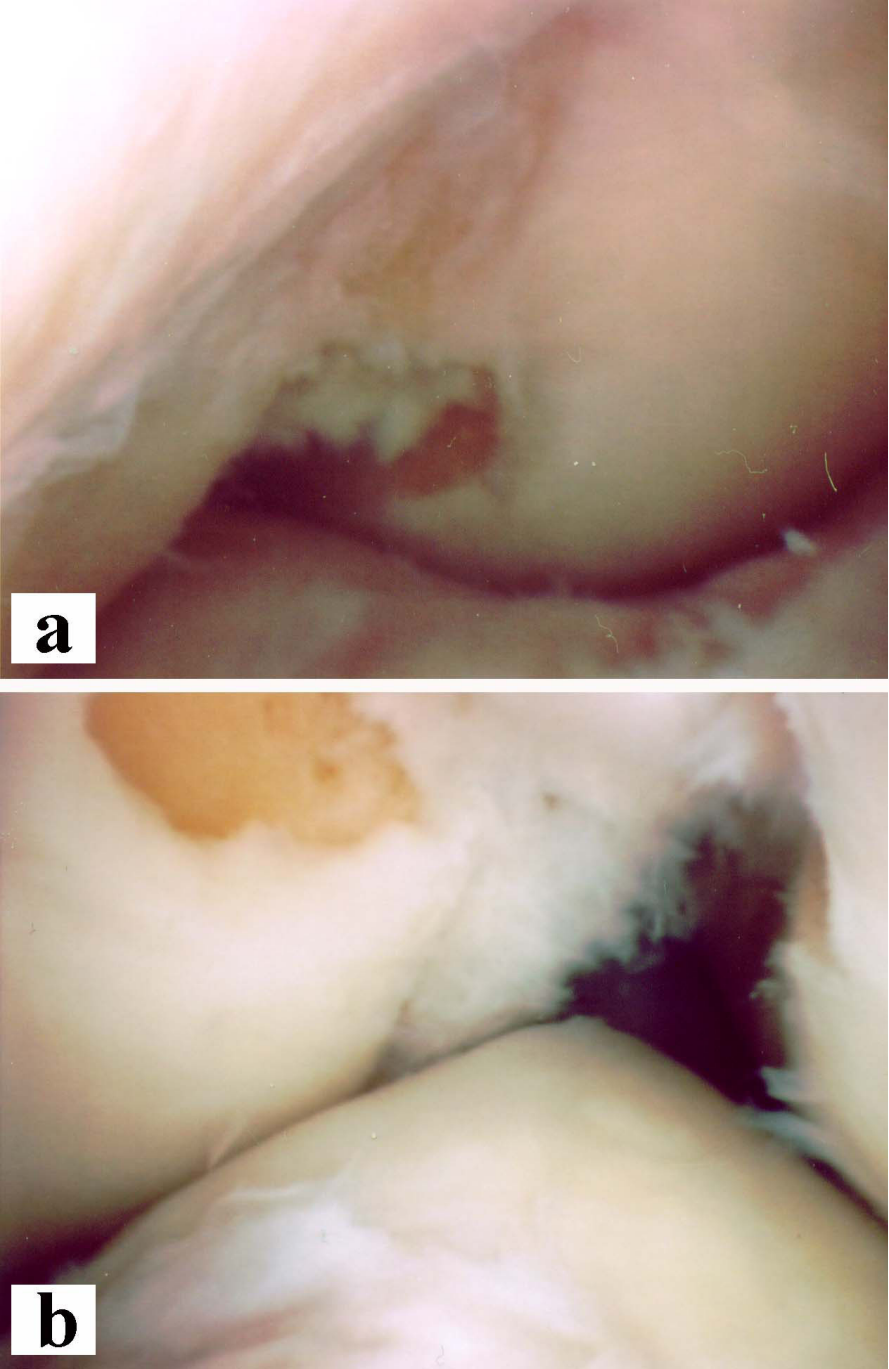
**Arthroscopy images**. Photographs obtained during arthroscopy of the right knee. Note the extremely narrow intercondylar notch (**a**) and the single tibial bump with a complete coverage with articular cartilage and a missing ACL footprint (**b**).

During exam under anesthesia the ligament findings were the same as during the clinical exam. Originally, it was planned to use ipsilateral and contralateral hamstrings as grafts. However due to abnormal appearance of the MRI (Figure 1a) it was decided to start with the diagnostic arthroscopy before tendon harvest on the contralateral side. At arthroscopy there was a hypoplasia of the medial trochlea, and a lateralization of the patella. The lateral compartment showed a small cartilage defect at the lateral femoral condyle. The trochleal groove revealed a bare bone region at the distal end as if it was an osteophytic bone formation in a chronic ACL case. The medial compartment was normal. The intercondylar notch was extremely narrow (Figure 2a). The tibia revealed a missing ACL footprint and a single bump with a complete coverage with articular cartilage (Figure 2b). The lateral condyle appeared to be enlarged. At the figure of 4 position a meniscofemoral ligament (MFL) could be identified connecting the posterior horn of the lateral meniscus to the medial femoral condyle. There was no room proximal to the MFL where an ACL graft would fit in. A PCL could not be identified from the view from anterior. The lateral meniscus appeared to be normal.

**Figure 3:**
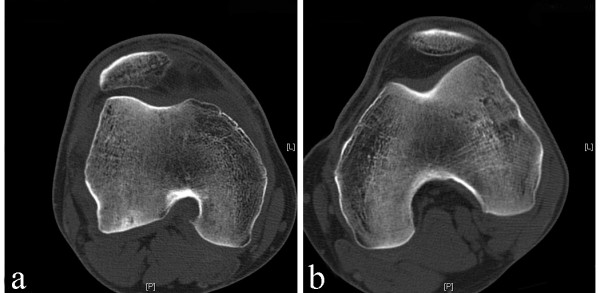
**Computed tomography scans**. Transversal layers of computed tomography scans of the affected (**a**) and contralateral (**b**) femur. Note the significant notch deformity in **a**.

**Figure 4:**
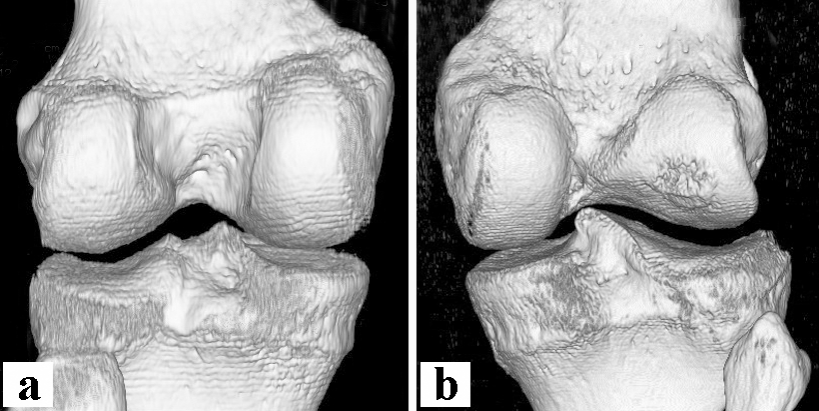
**Three-dimensional reconstruction of CT scans**. Posterior view on three-dimensional reconstruction of CT scans of the contralateral (**a**) and the affected (**b**) knee. **b **shows the extremely narrow notch and the deformity of the lateral femoral condyle. Note the malformation of the tibial eminence with only one spine (**b**) as opposed to the normal tibial eminence with two spines (**a**).

Due to the findings at surgery the procedure was ended since a ligament reconstruction did not appear possible in this case. Postoperatively the patient was informed on the unexpected aplasia and notch deformity making ligament reconstruction impossible. The patient underwent further evaluation with computed tomography scans (Figure 3) and three-dimensional reconstruction (Figure 4) to characterize the degree of bony deformity. The images affirmed the hypoplasia of the medial trochlea and the extremely narrow intercondylar notch. Three-dimensional reconstruction visualized the single tibial spine (Figure 4b) as opposed to usually two tibial spines in a healthy knee joint (Figure 4a).

The patient was further treated conservatively and did well at a reduced activity level at last follow up.

## Discussion

There are only few reports about aplasia or hypoplasia of the cruciate ligaments in the literature. Since patients are usually adapted to the congenital anatomy of their knee joints [3,14,15] laxity is most likely a coincidental finding after trauma [6,13,14]. Usually patients do not complain of instability, although clinical tests (e.g. Lachman, anterior/posterior drawer) are highly positive for ligament insufficiency [12,21-23]. The physician has to differentiate between objective laxity (positive tests for ligament insufficiency) and the subjective feeling of instability which is rarely reported by the patient.

Several radiological signs indicate aplasia of the cruciate ligaments. Common findings include hypoplasia of the tibial eminence [10,23,24], a hypoplastic lateral femoral condyle [19] and a narrow intercondylar notch [1,16].

Manner et al. recently published a study on the typical radiological findings of patients with arthroscopically proven aplasia of the cruciate ligaments [20]. They evaluated the associated pathological findings on MRI and tunnel view radiographs inaugurating a three stage classification system. According to their results the differentiation between trauma and aplasia of one or both cruciate ligaments may be made on plain radiographs according to differences in the notch width index, notch hight and changes in the lateral and/or medial tibial spine [20].

Our case demonstrates that the correct diagnosis may be missed in the clinical setup if a trauma is reported in the history and the contralateral knee is normal. A misleading information in this case was the previous arthroscopy report in which the specific finding of a severe notch deformity was not indicated. Also the external MRI report did not outline a deformity of the notch or the tibial spine. If the surgeon would have retrieved the contralateral hamstrings at the beginning of the planned ligament reconstruction procedure a significant damage would have occurred to the patient.

**Figure 5:**
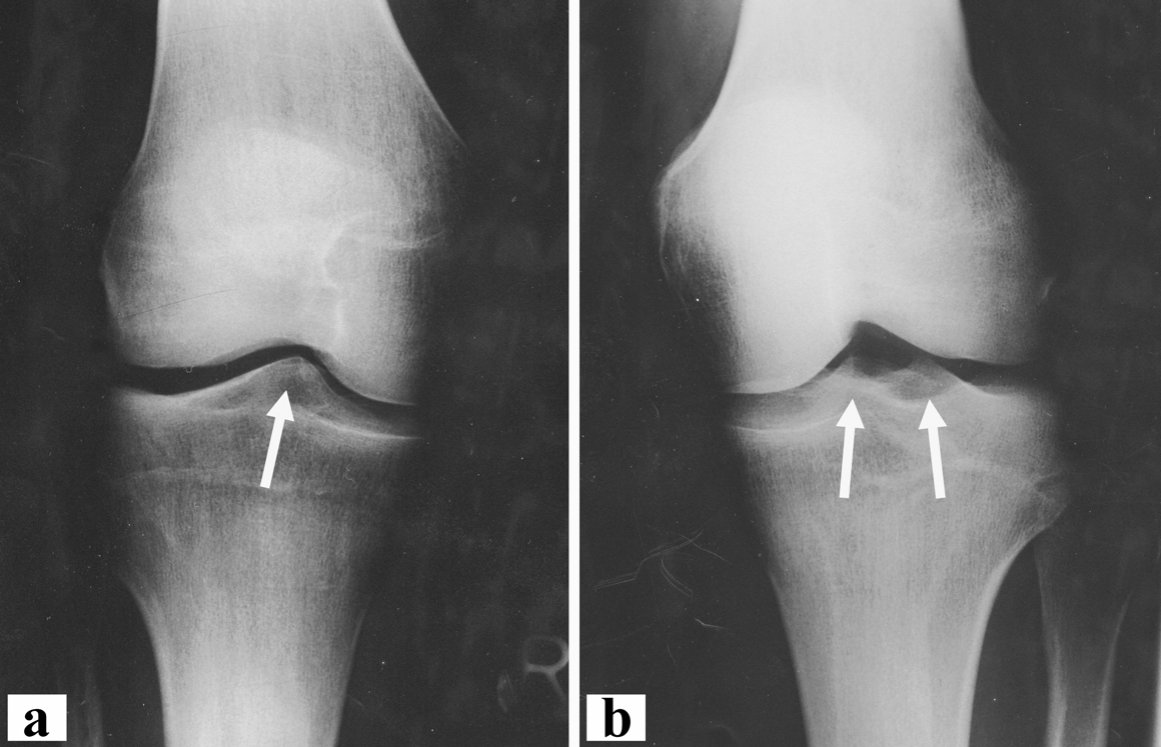
**Radiographs of both knee joints**. Anteroposterior radiographs of the affected (**a**) and contralateral (**b**) knee joint. The "dromedar-sign" with only one tibial spine is visible (**a **- arrow) as opposed to the normal radiological finding with two spines (**b **- arrows). If a "dromedar-sign" is visible on plain radiographs the arthroscopic surgeon should be alert of an aplasia of the cruciate ligaments.

In retrospect several pieces of information would have made the correct diagnosis possible prior to surgery. First looking at the MRI findings more closely would have revealed both notch and tibial spine abnormality (Figure 1a). Secondly, radiographs (Figure 5) revealed an abnormal tibial eminence with only one bump in the area of the tibial spine. We refer the abnormal finding of only one tibial spine to as a "dromedar-sign" (arrow in Figure 5a) as opposed to the two (medial and lateral) tibial spines in a normal knee (arrows in Figure 5b). This may be used as a hint for aplasia of the anterior and posterior cruciate ligaments.

The history of the patient about his early childhood revealed on a more closer look that there were signs of congenital abnormalities suggesting other abnormalities in the symptomatic knee.

In the literature therapeutical options are discussed controversially. Some authors report good results after ACL reconstruction and consider ligament insufficiency as a mechanical problem responsible for instability [3,12,13]. Others prefer conservative treatment with physiotherapy and muscular training [7,11,15,21,23]. If surgical treatment is taken into consideration, it should include reconstruction of both ligaments, since reconstruction of the ACL alone results in posterior subluxation of the tibia and a fixed posterior drawer causing decreased knee extension and anterior knee pain [22,25].

Ligament reconstruction in a case as described is technically hardly possible since there is no room in the knee for an additional ligament. A significant notch plasty if not a partial resection of one of the condyles would have been necessary to implant a cruciate ligament graft. However as this would be an absolutely arbitrary procedure it is most likely that this would not lead to a good knee stability.

## Conclusion

Even in seemingly clear diagnostic findings the arthroscopic surgeon should take this rare abnormality into consideration and be familiar with the respective radiological findings.

## Consent

Written informed consent was obtained from the patient for publication of this case report and any accompanying images.

## Competing interests

The authors declare that they have no competing interests.

## Authors' contributions

MB did the literature review and drafted the manuscript. JMH did all radiologic imaging and analysis. SS and JH performed the surgery and documentation of the case. DL helped to draft the manuscript and gave significant intellectual input. JH participated in the study design and coordination. All authors read and approved the final manuscript.
